# FBI-1 Enhances ETS-1 Signaling Activity and Promotes Proliferation of Human Colorectal Carcinoma Cells

**DOI:** 10.1371/journal.pone.0098041

**Published:** 2014-05-23

**Authors:** Min Zhu, Mingyang Li, Fan Zhang, Fan Feng, Weihao Chen, Yutao Yang, Jiajun Cui, Dong Zhang, Enqiang Linghu

**Affiliations:** 1 Department of oncology, Nan Lou Division, Chinese PLA General Hospital, Beijing, P. R. China; 2 Department of Gastroenterology, Nan Lou Division, Chinese PLA General Hospital, Beijing, P. R. China; 3 Tumor Center, Chinese PLA General Hospital, Beijing, P. R. China; 4 Department of Pharmacy, General Hospital of Shenyang Military Command, Shenyang, P. R. China; 5 Department of Urology, Chinese PLA General Hospital, Beijing, P. R. China; 6 Beijing Institute for Neuroscience, Capital Medical University, Beijing, P. R. China; 7 Department of Cancer and cell Biology, College of Medicine, University of Cincinnati, Cincinnati, Ohio, United States of America; 8 Department of Gastroenterology, Chinese PLA General Hospital, Beijing, P. R. China; Northwestern University, United States of America

## Abstract

In this study, we investigated a potential regulatory role of FBI-1 in transcription factor activity of ETS-1. The protein interaction was identified between ETS-1 and FBI-1 in lovo cells. The accumulating data showed that FBI-1 promoted the recruitment of ETS-1 to endogenous promoter of its target genes and increase ETS-1 accumulation in the nuclear. Our work also indicated that the FBI-1 enhances ETS-1 transcription factor activity via down-regulating p53-mediated inhibition on ETS-1. Further, FBI-1 plays a role in regulation of colorectal carcinoma cells proliferation. These findings supported that FBI-1 might be a potential molecule target for treating colorectal carcinoma.

## Introduction

Human pro-oncogene FBI-1 is a master transcriptional regulator that belongs to the POK protein family [1]. It has been known by several names such as LRF (leukemia/lymphoma-related factor) [2] or Pokemon (POK erythroid myeloid ontogenic factor) [3]. FBI-1, characterized by the BTB/POZ domain, plays an important role in the regulation of development and progression of various cancers, like breast cancer, hepatocellular carcinoma and leukemia [4]. The POZ domain proteins modulate various cellular regulatory functions [5]. Members of POZ family would repress gene transcription via their POZ domain. Some of the POK proteins also contain a Krüppel like zinc finger domain [5]. In human cancerous cells, FBI-1 would repress p53 signaling activity through regulating Hdm2, Mdm2, and p19^ARF^, p21 and Rb expression [1], [6]–[8]. Recently, Fang et al reported that FBI-1 promoted HCC cells proliferation [4], Cui et al reported that FBI-1 interacted with AR in LNCaP cells [5]. Moreover, in AR-independent prostate cancer cells, FBI-1 acts as a positive cell proliferation regulator. Based on the evidence that the POZ domain would mediate the protein interaction of FBI-1 with transcription factors [5], we examined the interaction of FBI-1 with some other transcriptional factors (Data not shown). The results showed that FBI-1would interact with ETS-1, which plays certain roles in cancerous cells proliferation, migration and invasion. So, FBI-1 would also function via ETS-1. It is valuable to declare the interaction between FBI-1 and ETS-1.

ETS-1 is characterized by the helix DNA-binding domain and the ETS domain (transcription activation domain) [9]. The members of ETS family are involved in the regulation of cell development, differentiation, proliferation, apoptosis, migration, tissue remodeling, invasion and angiogenesis [10]–[11]. Among ETS family proteins, ETS-1 is highly expressed in breast cancer, ovary cancer, and cervical carcinomas and is associated with a poor prognosis [12], [13]. In nucleus, ETS-1 binds to the ETS-binding sequences in the promoter/enhancer regions of their target genes, such as MMP1, MMP9, u-PA and c-Met [9]–[12]. The sequence of ETS response element is 5′-GGAA/T-3′ [10]–[12], [14]. The activity of ETS-1 is regulated by some co-regulators, such as SRC-1, AIB-1 and NCoR [14], [15]. Granted that ETS-1 plays an important role in multiple cancers,it is valuable to identify more regulators of ETS-1.

Human colorectal carcinoma may be one of the most intractable cancers in nowadays [16]. In this study, we found that FBI-1 interacts with ETS-1. Overexpression of FBI-1 enhanced ETS-1 transcription factor activity, whereas knockdown of endogenous FBI-1 significantly inhibited ETS-1 transcriptional activity. Multiple lines of evidence suggested that FBI-1 functions through promoted the recruitment of ETS-1 to the its downstream genes' promoter and enhanced the accumulation of ETS-1 in the nuclear. P53 is participated in the effect of FBI-1 on ETS-1 activity. Moreover, FBI-1 would promote human colorectal carcinoma cells LoVo, HR8348, and HT29 growth. Furthermore, FBI-1 enhanced the invasion and anchor-independent growth ability of Lovo cells.

## Materials and Methods

### Plasmids

The FBI-1, FLAG-FBI-1expression vectors, FBI-1 siRNA expression vector and ARE-Luc are from reference [3], [5]. The cDNA sequence of ETS-1 was cloned into pIRES vector linked with FLAG at amino terminus by PCR, using cDNA library (Invitrogen, Carlsbad, CA) as template. The Small interfering RNA (siRNA) against p53 was purchased from Cell Signaling Technology following reference [17]. The siRNA vector targeted to ETS-1 was purchased from Santa Cruz Biotechnology. Luciferase reporter (EBS-Luc): EBS (ETS binding site) (GGAA) _8_ sequence was synthesized by using chemical synthesis methods (Gene Ray Company, Shanghai, China). All of the vectors were confirmed by DNA sequencing.

### Antibodies

Antibodies against ETS-1, P53, MMP1, MMP9, u-PA, c-Met, and GAPDH were from Santa Cruz Biotechnology, USA and antibodies against FBI-1 from Sigma Technology, St. Louis, USA. A polyclonal anti-rabbit IgG antibody and monoclonal anti-Flag monoclonal antibody both conjugated with the HRP (horseradish peroxidase, HRP) were from Sigma, St. Louis, USA.

### Stable transfection

Plasmids were transfected into cells by using the Lipofectamine 2000 (Invitrogen, Carlsbad, CA). Transfected cells were cultured in 500 µg/ml G418 (Invitrogen, Carlsbad, CA) for approximately 1–2 months. Then individual clones were screened by Western blot assays. Similar results were observed with stable transfection or transient transfection, individual clone or pool clone.

### Luciferase assay

Human colorectal carcinoma cell Lovo, which stably transfected with plasmids, was seeded in 24 wells plate (corning, NY, USA) DMEM medium (Gibco, USA) supplemented with 10% charcoalstripped FBS (Hyclone, USA). Transfections were performed by the Lipofectamine 2000 agent (Invitrogen, Carlsbad, CA). Cells were co-transfected with Luciferase reporters and harvested for the luciferase and β-galactosidase activities analysis [18], [19]. The luciferase assays were performed for three independent times with similar results.

### Immunoblotting analysis

Lovo cells were seeded and cultured in 6-well plates. Then cells were transfected with plasmids and harvested after 48 h. Total protein samples were performed by SDS-PAGE and trans-printed to the NC membrane (Roche, Germany). NC Membranes were blocked with 10% BSA in TBST buffer and incubated with antibodies. Then membranes were incubated with the HRP-conjugated secondary antibodies after washed by TBST buffer for 3 times. At last, membranes were visualized by using the kit (Qiangen, Beijing, China). The blots were performed for three independent times with similar results. When incubating HRP-Flag monoclonal antibody, the blots were visualized without incubating secondary antibody.

### Immunoprecipitation

Lovo cells (American Type Culture Collection, ATCC) were transfected with expression plasmids using the Lipofectamine 2000 agent (Invitrogen, USA). Cells were harvested and lysed in the IP buffer after cultured for 24 h. The Co-IP analyze was performed with anti-FLAG monoclonal antibody (Sigma-Aldrich, St. Louis, USA) and then detected by the IB exam.

### Chromatin immunoprecipitation

The Chromatin immunoprecipitation (ChIP) assay was performed following a protocol provided by the ChIP kit (Upstate, USA). Lovo cells, which were stably transfected with plasmids, were fixed by adding formaldehyde to the medium to a final concentration of 1%. After cross-linking, glycine was added to a final concentration of 125 mM, and the cells were then harvested with lysis buffer. The nuclei of the cells were pelleted by centrifugation and re-suspended in nuclear lysis buffer. The nuclear lysates were sonicated to generate to the DNA fragments size of 0.5–1 kb, and then the immunoprecipitation assay was performed with anti-ETS-1 or anti-FBI-1 antibodies, respectively. Real-time PCR amplification was performed with DNA extracted from the immunoprecipitates and primers flanking the ETS-1 response elements in the MMP1 promoter. The primers used, MMP1, MMP9, or c-Met promoter, are as follows:

Input Genomic DNA forward: 5′-AACCTATTAACTCACCCTTGT-3′

Input Genomic DNA reverse: 5′-CCTCCATTCAAAAGATCTTATTATTTAGCATCTCCT-3′

MMP1 promoter forward: 5′- TTCCAGCCTTTTCATCATCC-3′

MMP1 promoter reverse: 5′- CGGCACCTGTACTGACTGAA-3′

MMP9 promoter forward: 5′- TACATTGGTACCTCTTGGGTCTTGGCCTTAGT -3′

MMP9 promoter reverse: 5′- TTGATACTCGAGCCAGCACCAGGAGCACC -3′

c-Met promoter forward: 5′- TCAAGTTCTAACCGCAATGC -3′

c-Met promoter reverse: 5′- AGAGGCCGAGAGCAAAGCTC-3′

### Immunocytochemistry

The Lovo cells were cultured on a glass, fixed with 3% paraformaldehyde for 30 min, and permeablized by Triton X-100 treatment for 10 min at 4°C. After blocking by 10% goat serum (PBS), the slices were incubated with primary antibody (anti-ETS-1) at 4°C for more than 4 hr, followed by washing thrice with PBS and incubation with FITC-labeled secondary antibody (goat anti-rabbit) for an additional 2 hr at room temperature while keeping from light. The nucleuses were stained by DAPI for 10–15 min at room temperature, and the fluorescence signals were visualized by a confocal microscope.

### Subcellular fractionation

The effect of FBI-1 on ETS-1 localization was determined by subcellular fractionation following reference [20]. Briefly, Lovo cells, which were stably transfected with plasmids, were homogenized using a Dounce homogenizer, and the homogenate was centrifuged at 600 rpm for 10 min. The pellet was analyzed as the nuclear fraction whereas the supernatant was centrifuged again at 20000 rpm for 10 min, and the final supernatant was analyzed as the cytoplasmic fraction.

### Cell culture and cell proliferation assays

Lovo, HR8348 and HT29 cells (American Type Culture Collection, ATCC) were cultured in DMEM (GIBCO, USA) Medium with 10% FBS. Then, cells were seeded in 96-well cell culture plates. After incubating for 1 day, 2 day, 3 day and 4 day, Cells were harvested. The MTT Cell growth assays were performed for three independent times with similar results [20].

### Anchorage-independent growth assay

Lovo Cells were stably transfected with plasmids. Then, cells (500 per well) were plated on 6-well plates (corning, NY, USA), with a bottom layer of 0.7% low-melting-temperature agar in DMEM and a top layer of 0.25% agar in DMEM. Colonies number are the mean ± SE of three independent experiments scored after 3–4 weeks of growth [19].

### Statistical analysis

Statistical significance in the luciferase activity and cell growth assays was analyzed by Bonferroni correction with or without two-way ANOVA.

## Results

### FBI-1 modulates the transcriptional activity of ETS-1 in Lovo cells

**Figure 1:**
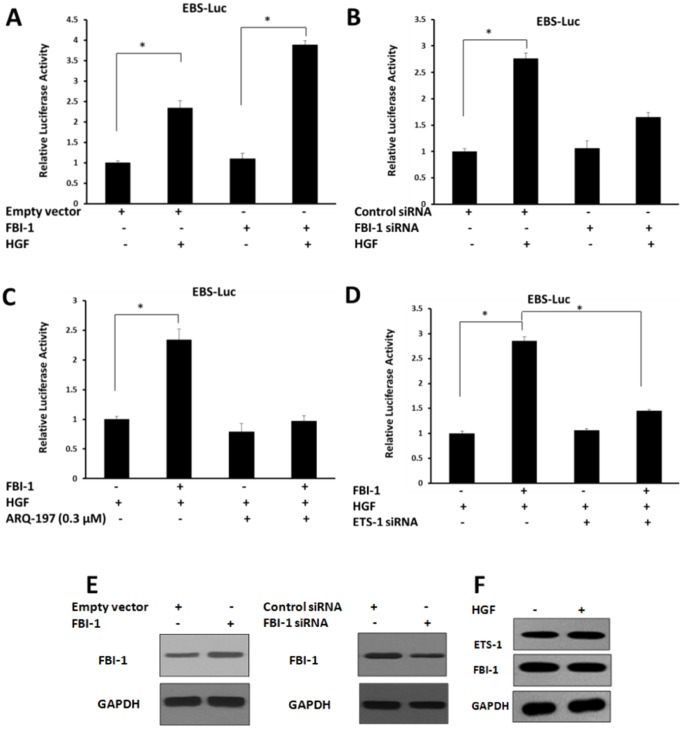
FBI-1 enhances transcriptional activity of ETS-1. (A-D) Lovo cells were transfected with ETS-RE-Luc reporter. (A, C-D) Cells were stably transfected with FBI-1 expression vector or empty vector. (B) Cells were transfected with FBI-1 siRNA or control siRNA. (D) Cells were transfected with ETS-1 siRNA (D). Lovo cells were treated with 5 ng/ml HGF (A-D, F), or ARQ-197 (C). (E) Overexpression or knockdown of FBI-1 were examined by western blot. The luciferase values are the mean ± SE of three independent experiments with similar results. *P<0.05 versus with the empty vector or the FBI-1 vector (A-D); versus with the control siRNA or the FBI-1 siRNA (A-D); versus with or without HGF/ARQ-197.

To define the role of FBI-1 in the activity ETS-1, luciferase assays were performed. The activity of luciferase reporter EBS-Luc (ETS-1 binding site luciferase reporter) was measured to reflect ETS-1 activity. As shown in Fig. 1A-B, FBI-1 enhanced the transcription activity of ETS-1 in the presence of HGF. To determine if endogenous FBI-1 may be involved in ETS-1 transactivation, the siRNA targeted to FBI-1 was used to diminish FBI-1 protein level. Reduction of FBI-1 expression decreased ETS-1 transcriptional activity for 2-folds in response to HGF. At the same time, c-MET inhibitor ARG-197 and ETS-1 siRNA were used. FBI-1 could not enhance the ETS-1 activity in presence of ARG-197 or ETS-1 siRNA (Fig. 1C-D). Over-expression or knockdown of FBI-1 was examined by western blot (Fig. 1E).

Next, we examined the effect of HGF on FBI-1 and ETS-1 expression. The results showed that HGF did not significantly increased FBI-1 and ETS-1 expression (Fig. 1F). This suggested that FBI-1 enhanced ETS-1 transcriptional activity in Lovo cells. At the same time, HGF is the agonist of HGF/c-Met/ETS-1 signaling, and ARQ-197 is the antagonist of HGF/c-Met/ETS-1 signaling. The expression of FBI-1 or ETS-1 would not be affected by HGF after 24 hour treatment. FBI-1 would promote HGF/c-Met/ETS-1 activity via interacting with ETS-1.

### FBI-1 increases the expression of ETS-1 targeted genes

**Figure 2:**
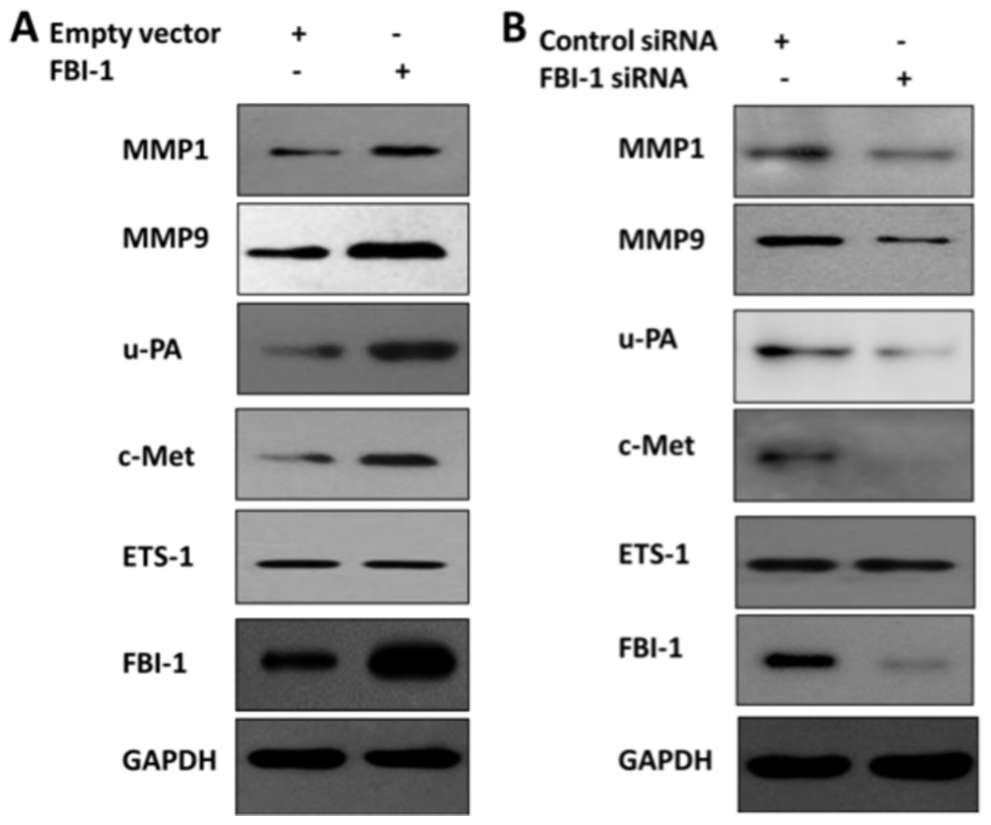
FBI-1 modulates ETS-1 downstream genes expression in Lovo cells which were stably transfected with plasmids. (A-B) Western blotting with various antibodies showed the overexpression of FBI-1 or specific knockdown effect of FBI-1 siRNA on the endogenous FBI-1 protein level. Lovo cells were harvested for WB assays and detected by anti-FBI-1 antibody, anti-MMP1 antibody, anti-MMP9 antibody, anti-u-PA antibody, anti-c-Met antibody, anti-ETS-1 antibody, or anti-GAPDH antibody.

To confirm whether FBI-1participates in regulation of the ETS-1 downstream genes expression, immunoblotting (IB) assays were performed. As expected, overexpression and knock-down of FBI-1 protein level did not affect the ETS-1 expression (Fig. 2A and B). Our results also showed that FBI-1 increased the expression of u-PA, c-Met, MMP1 and MMP9 (Fig. 2A). Reduction of the FBI-1 expression with FBI-1 siRNA decreased the protein level of u-PA, c-Met, MMP1 and MMP9 (Fig. 2B). All the luciferase and IB results suggested that FBI-1 may increase the expression of ETS-1 response genes in Lovo cells.

### Interaction of FBI-1 with ETS-1 and p53

**Figure 3:**
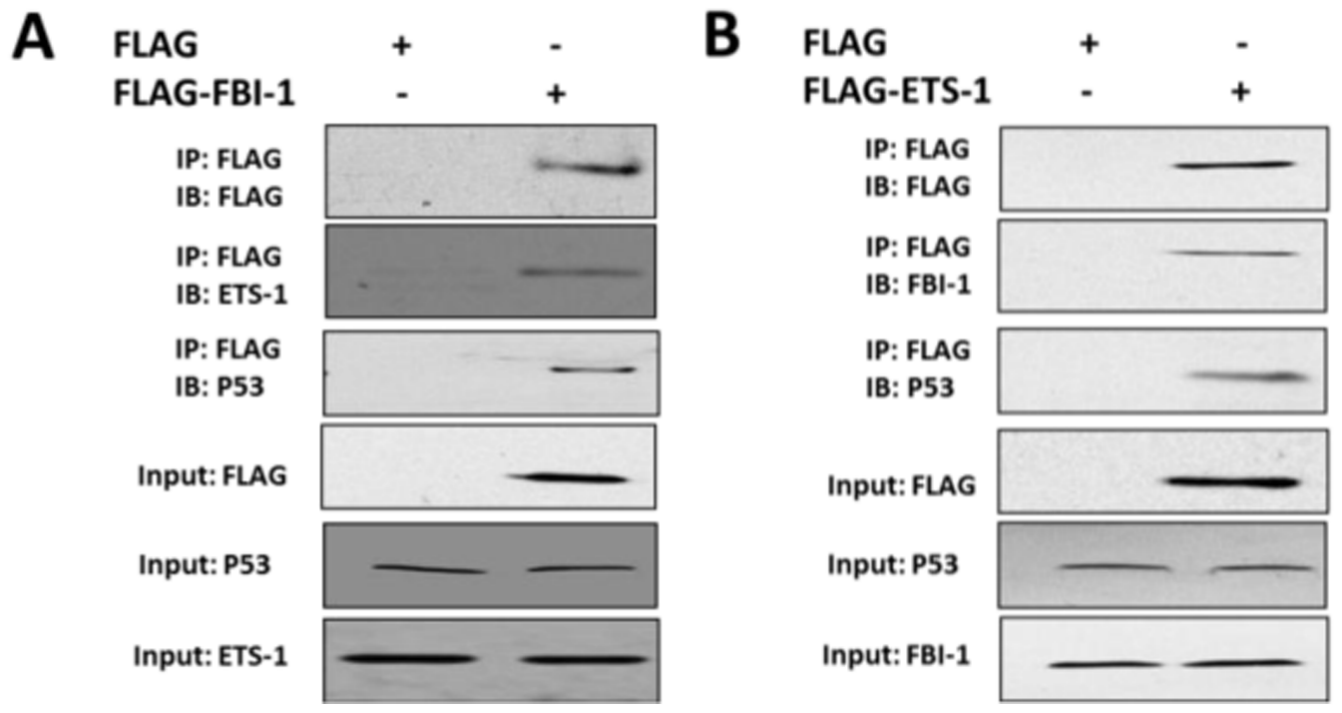
FBI-1 would potentially interact with ETS-1 and P53 in vivo. (A) Interaction of FLAG-FBI-1 with exogenous ETS-1 or p53 in vivo. Lovo cells were transfected with FLAG-tagged FBI-1 or FLAG empty vector. Then, cell lysates were immunoprecipitated (IP) by anti-FLAG beads, and the precipitates were then immunoblotted (IB) with anti-FLAG antibody, anti-ETS-1 antibody, or anti-p53 antibody. (B) Lovo cells were transfected with FLAG-ETS-1 vector or FLAG empty vector. The IP analysis was performed with anti-FLAG antibody, and the IB analysis was performed with anti-FLAG antibody, anti-FBI-1 antibody, or anti-P53 antibody.

Next, we examined the possible interaction between FBI-1, ETS-1 and p53. As shown in Fig. 3A, FLAG-FBI-1 but not FLAG interacted with endogenous p53 and ETS-1. Furthermore, the Re-IP assay was Performed (Fig. 3B). FLAG-ETS-1 interacted with p53 and FBI-1. Taken together, FBI-1, p53 and ETS-1 may potentially form a protein complex. In addition, p53 only interacts with ETS-1 but not affect the protein level of ETS-1 (Fig. S1 in File S1).

### FBI-1 modulates the recruitment of ETS-1 to MMP1 promoter

**Figure 4:**
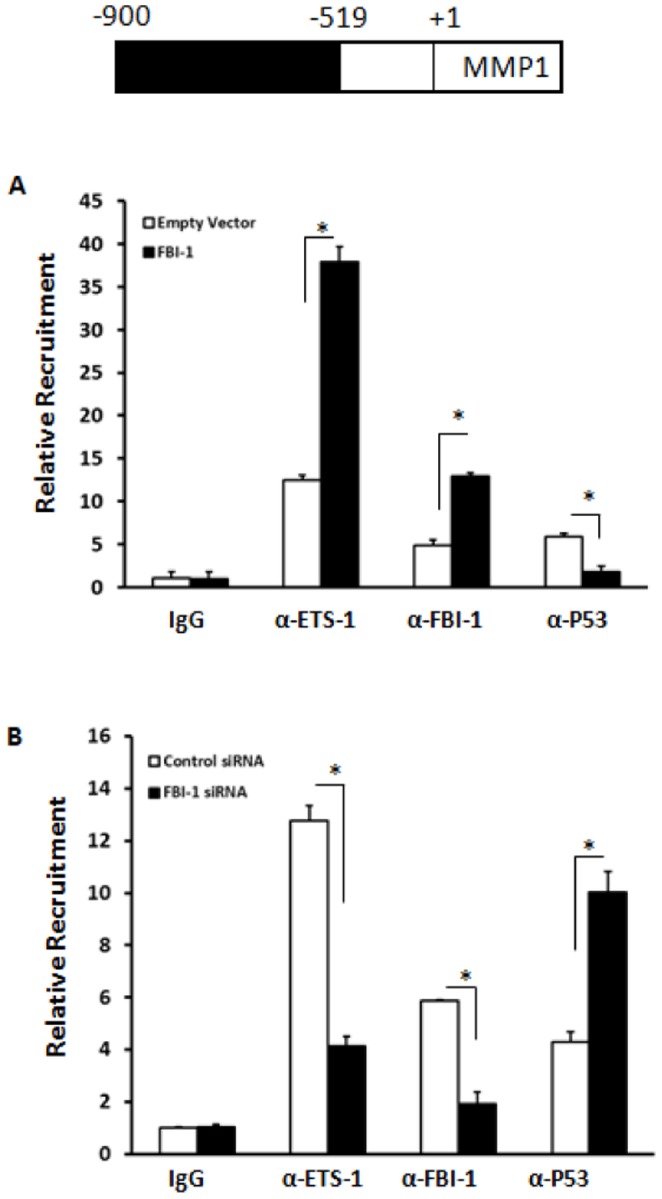
FBI-1 can enhance the recruitment of ETS-1 to the MMP1 promoter. (A) Lovo cells stably transfected with FBI-1 or empty vector were prepared and subjected to ChIP by using IgG antibody (negative control) or antibodies for ETS-1, FBI-1 and p53. The Immunoprecipitated DNA fragment was quantified by real-time PCR assay. (B) Lovo cells, which were stably transfected with FBI-1 siRNA, or control siRNA, were harvested for the ChIP assays. The ChIP assays were performed with IgG antibody (negative control) or antibodies for ETS-1, FBI-1 and p53. *P<0.05 versus the empty vector or the FBI-1 vector (A); or versus the control siRNA or the FBI-1 siRNA (B). The cloned promoter region of MMP1 is showed above the figure.

The ChIP assay was conducted to investigate the possibility that FBI-1 could affect the recruitment of ETS-1 to the endogenous MMP1 promoter. The results showed that FBI-1 significantly promoted the recruitment of ETS-1 to the MMP1 promoter sequence (Fig. 4A) whereas knock down of endogenous FBI-1 protein level via siRNA inhibited the recruitment of ETS-1 on MMP1 promoter (Fig. 4B). The specificity of the recruitment was examined by control IgG. The IgG failed to IP the MMP1 promoter fragment (Fig. 4A and B). These suggest that FBI-1 enhanced the ETS-1 transcriptional activity by enhancing the recruitment of ETS-1 to endogenous MMP1 promoter. It is reported that p53 and ETS-1 interacts in cancerous cells [23]–[25]. Our results also showed that P53 would also occupancy at the MMP1 promoter which contains the ETS-1 response element (Fig. 4A–B). FBI-1 inhibited the recruitment of P53 on MMP1 promoter (Fig. 4A–B).

Next, the recruitment of ETS-1, FBI-1 and P53 to some other ETS-1 targeted genes. The results showed that overexpression of FBI-1 promoted the recruitment of ETS-1 to MMP9 (Fig. S2 in File S1) and c-Met promoter sequences (Fig. S3 in File S1). Down-regulation of FBI-1 expression via its siRNA disrupted the recruitment of ETS-1 to MMP9 and c-Met promoter sequences.

### Effect of FBI-1 on ETS-1 sub-cellular localization

**Figure 5:**
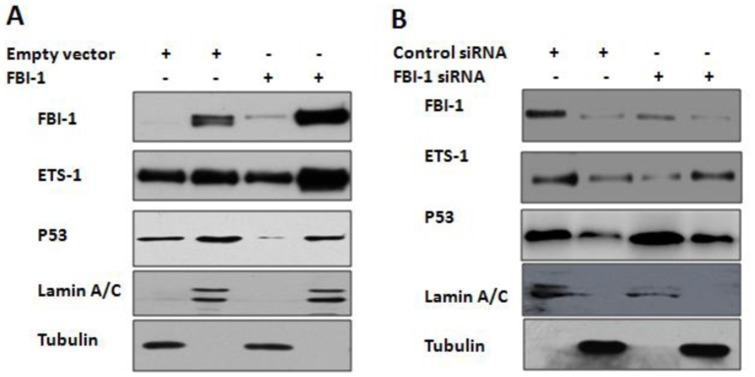
Effect of FBI-1 on ETS-1 cytoplasmic/nucleus localization. (A–B) The cells were fractionated into cytoplasmic and nuclear fractions. The fractions were examined with anti-ETS-1 antibody, anti-P53 antibody and anti-FBI-1 antibody. The Lamin A/C and tubulin were used as the nuclear and cytoplasmic indicator, respectively.

To investigate whether FBI-1 modulates ETS-1 activity could be due to cytoplasmic/nucleus trans-location, we performed the subcellular fractionation assay, followed by IB analysis with the anti-ETS-1 antibody. As shown in Fig. 5, ETS-1 and P53 could be detected both in cytoplasmic and nuclear. As expected, overexpression of FBI-1 promoted the nuclear accumulation of ETS-1 (Fig. 5A) and knockdown of FBI-1 expression inhibited this translocation (Fig. 5B). In addition, FBI-1 reduced the translocation of P53 from cytoplasmic to nuclear (Fig. 5A and B). Next, the localization of ETS-1 protein was examined by immunocytochemistry assay. Overexpression of FBI-1 promoted the nuclear accumulation of ETS-1 (Fig. S4A-B in File S1) and knockdown of FBI-1 expression inhibited this translocation (Fig. S4C-D in File S1). These data suggest that FBI-1 enhanced ETS-1 activity would due to promote the nuclear accumulation of ETS-1 and inhibit P53 cytoplasmic/nucleus translocation.

### P53 participates in enhancement of FBI-1on ETS-1 activity

**Figure 6:**
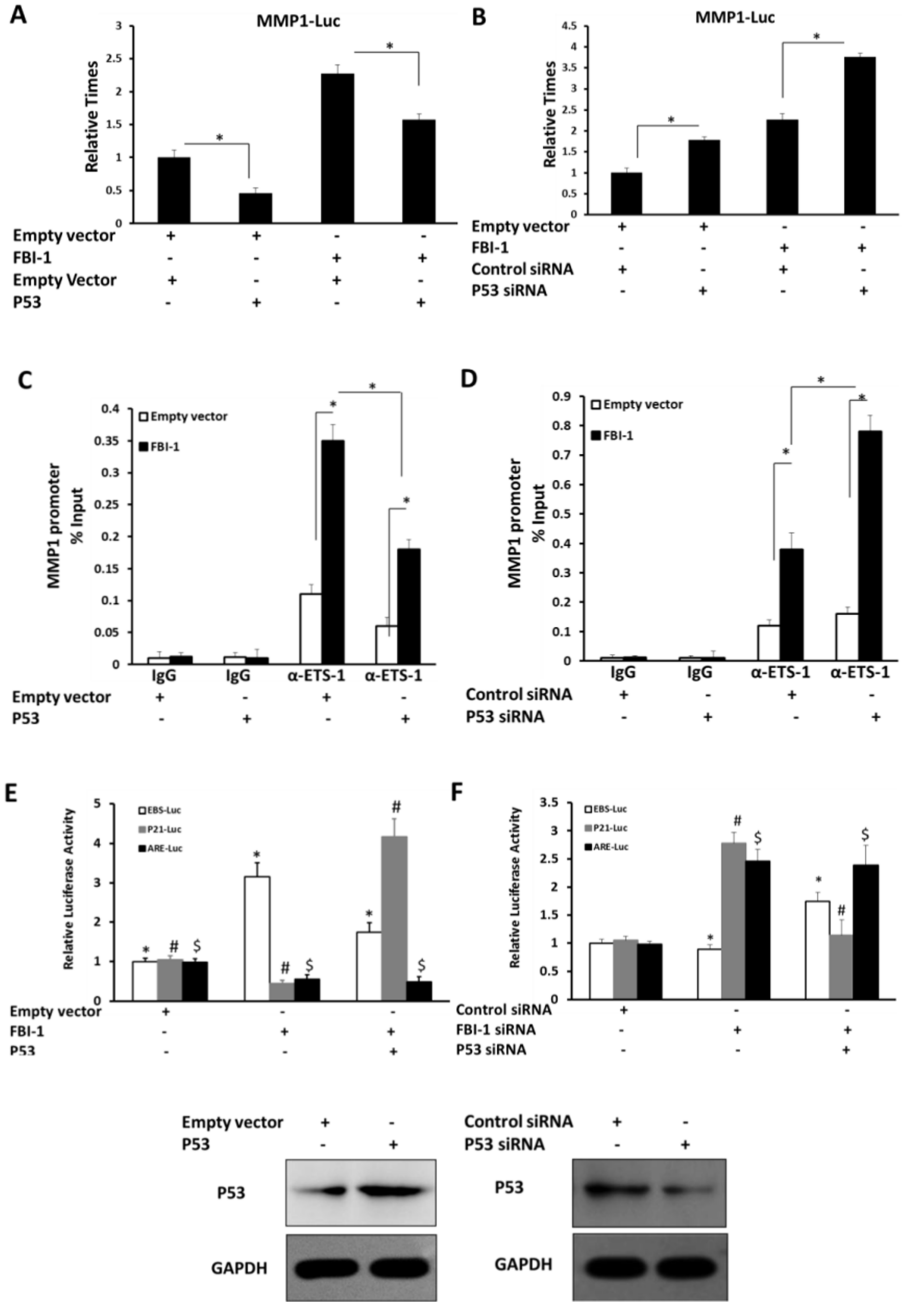
p53 participates in FBI-1 modulating ETS-1 activity. (A–D) Lovo cells were transfected with FBI-1 expression vector or empty vector. (A–B) Lovo cells were co-transfected with MMP1-Luc vector, p53 siRNA, or control siRNA, p53 vector or empty vector as indicated. Following transfection, cells were harvested for the luciferase assay. The values are the mean ± SE of three independent experiments with similar results. (C–D) Lovo cells were co-transfected with p53 vector or empty vector, p53 siRNA or control siRNA. The ChIP assays were performed with IgG of anti-ETS-1 antibody. (**E–F**) The luciferase activities changing of EBS-luc, p21-luc and ARE-luc upon FBI-1 and p53 overexpression and knocking down. The values are the mean ± SE of three independent experiments with similar results. *P<0.05 versus the empty vector or the FBI-1 vector (A–D), *P<0.05 versus the empty vector or the p53 vector (A, C), *P<0.05 versus the p53 siRNA vector or the control siRNA vector (B, D).

The IP results indicated that FBI-1, p53 and ETS-1 potentially formed a protein complex. It is necessary to test the involvement of p53 in the enhancement of ETS-1 activity by FBI-1. Lovo cells, which were stably transfected with FBI-1 expression vector, or empty vector, were co-transfected with p53 vector, or empty vector, control siRNA or p53 siRNA. As shown in Fig. 6A and B, the overexpression of FBI-1 increased ETS-1 activity and recruitment to MMP1 promoter. Overexpression of p53 markedly inhibited the effect of FBI-1on ETS-1 (Fig. 6A). Knockdown of p53 protein level effectively promoted the effect of FBI-1 (Fig. 6B). However, FBI-1 can partially affect the ETS-1 transcriptional activity and recruitment to MMP1 promoter in the presence of siRNA against p53 (Fig. 6A and B). Next, the involved of p53 on FBI-1 function were determined. As shown in Fig. 6 C and D, p53 diminished the effect of FBI-1 on ETS-1 recruitment. This result suggests that the interaction of P53 would partially participate in the FBI-1 effect on ETS-1 activity. When FBI-1 was over-expressed, the activity of EBS-luc was elevated. But when p53 was co-overexpressed, this effect was weakened. Knock-down of FBI-1 or both p53 and FBI-1 showed the opposite result of EBS-luc activity, which supported the over-expression experiment. On the other hand, activities of the p53 specific responsible reporter vector p21-luc and FBI-1/ETS-1 specific responsible reporter vector ARE-luc could not be rescued when p53 was co-knockdown or co-overexpressed with FBI-1 (Fig. 6E and F).

### FBI-1 enhances colorectal carcinoma cells growth

**Figure 7:**
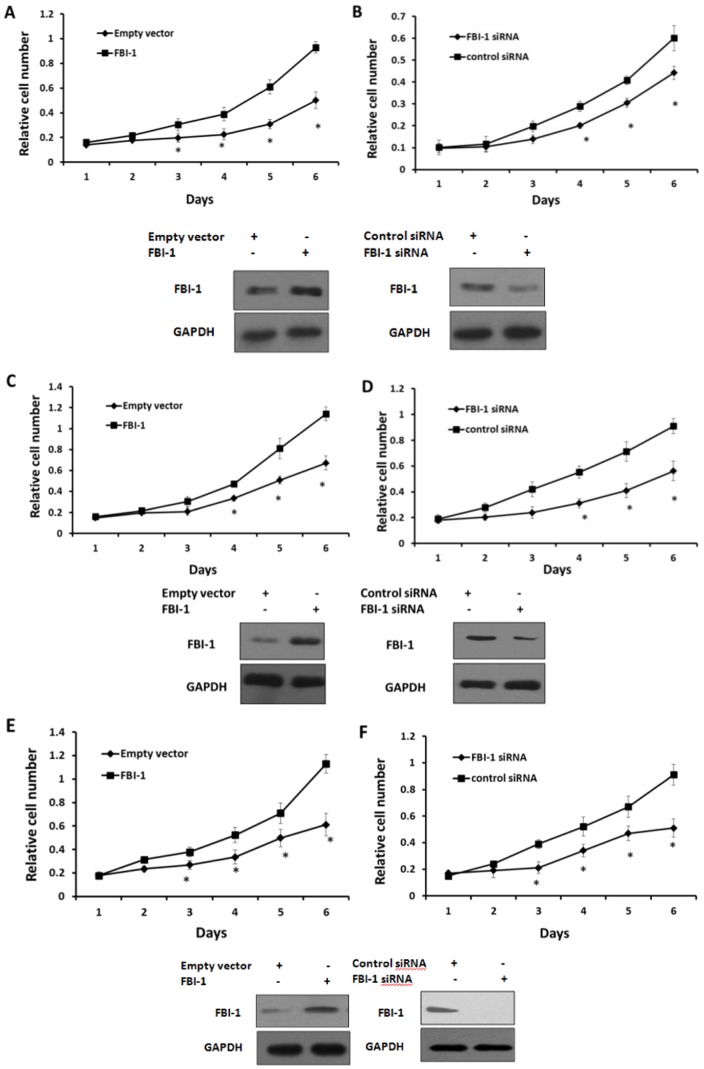
FBI-1 promotes colorectal carcinoma cells proliferation in vitro. (A–B) LoVo, (C–D) HR8348, and (E–F) HT29 cells were stably transfected with the plasmids. Then, relative cell numbers were determined by the MTT assay. Relative cell numbers (A and B) shown are Mean± SD of triplicate measurements and have been repeated 3 times with similar O.D. value results. *P<0.05 versus the empty vector or the FBI-1 vector (A, C, E), *P<0.05 versus the FBI-1 siRNA vector or the control siRNA vector (B, D, F).

To delineate the biologic function and regulatory effect of FBI-1 on the colorectal carcinoma cells, MTT assays were performed. Overexpression of FBI-1 markedly promoted LoVo, HR8348, and HT29 cells growth (Fig. 7A, C and E); whereas reduction of FBI-1 protein level inhibited LoVo, HR8348, and HT29 (Fig 7B, D and F) cells growth. These data suggested that FBI-1 would enhance the proliferation ability of colorectal carcinoma cells.

### FBI-1 promoted colorectal carcinoma cell Lovo anchorage independent growth

**Figure 8:**
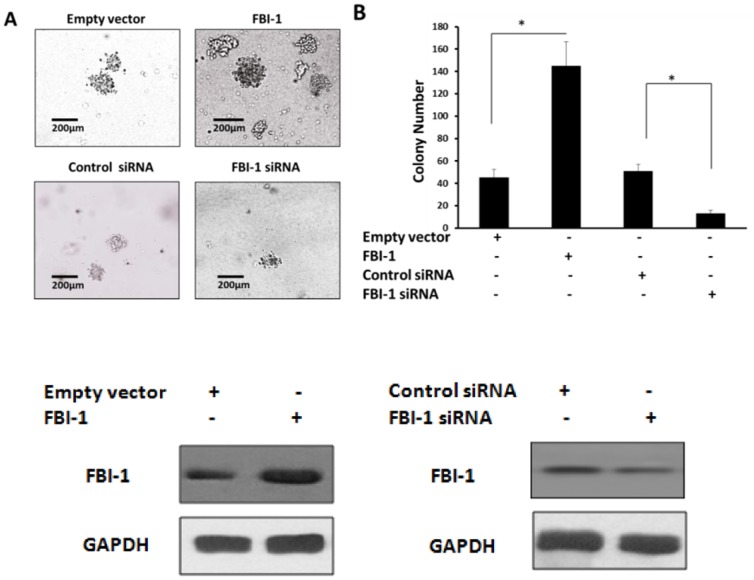
FBI-1 promotes Lovo cells anchor-independent growth and invasion. (A–B) Lovo cells were stably transfected with the FBI-1 expression vector or the empty vector, or the FBI-1 siRNA vector or the control siRNA vector. Colony number was shown in the photographs (B).*P<0.05 versus the empty vector or the FBI-1 vector (A–B), *P<0.05 versus the FBI-1 siRNA vector or the control siRNA vector (A–B).

To further confirm the critical roles of FBI-1 in human colorectal carcinoma cell, soft agar assay was performed. Lovo cells were stably transfected with plasmids. Overexpression of FBI-1 significantly increased Lovo cells anchorage independent growth ability (Fig. 8A and B), whereas the siRNAs targeted to the FBI-1 reduced Lovo cells anchorage independent proliferation (Fig. 8A and B). The soft agar results suggested that FBI-1 would promote Lovo cells metastasis.

## Discussion

**Figure 9:**
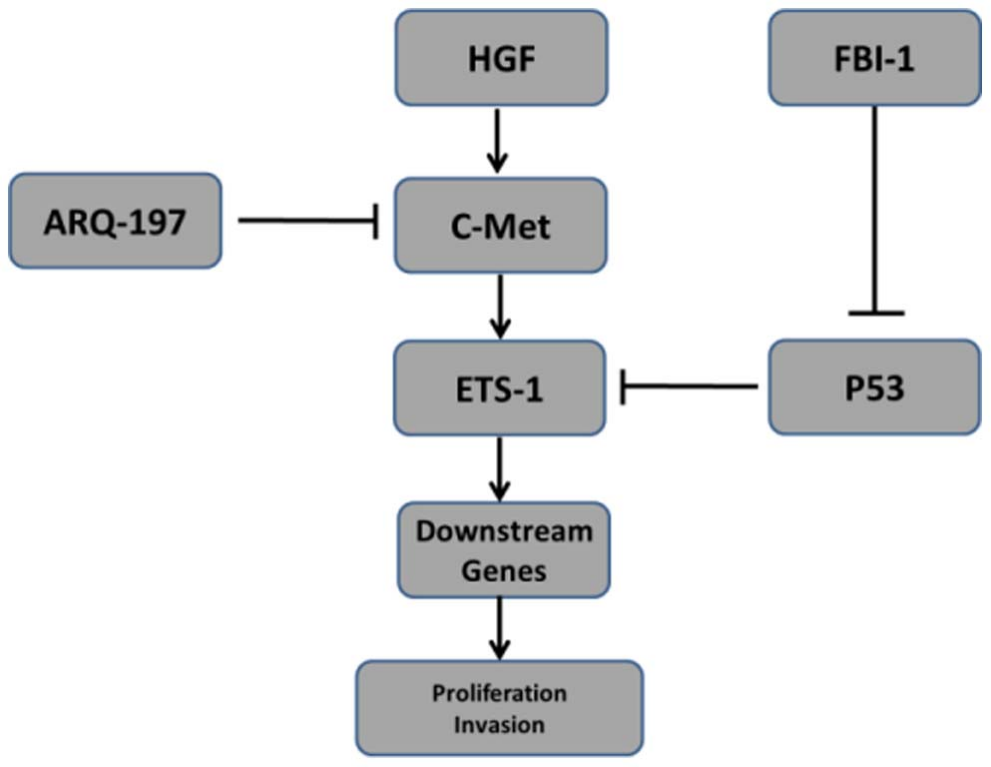
The proposed models for certain roles of FBI-1 function in Lovo cells. ETS-1 would be activated in presence of HGF and be blocked by ARQ-197. FBI-1 may modulate ETS-1 activity through potential protein interaction, recruitment to endogenous MMP1 promoter, or cytoplasmic/nucleus translocation. The regulatory effect of FBI-1 on ETS-1 is also through regulating P53 activity.

In this study, we provide evidence for the novel role of FBI-1 in colorectal carcinoma cells. Overexpression of FBI-1 enhanced ETS-1 activity, whereas knockdown of endogenous FBI-1 via its siRNA significantly reduced ETS-1 activity. FBI-1 potentially interacted with ETS-1 in lovo cells. Multiple lines of evidence suggested that FBI-1 modulated ETS-1 activity via its recruitment to downstream genes promoter, accumulation in nuclear. The P53, FBI-1 and ETS-1 might form a protein complex. Moreover, FBI-1 promoted the proliferation of the colorectal carcinoma cells LoVo, HR8348, and HT29. Given that HT29 carries mutant p53 (Arg273His), while Lovo express wild type p53, it seems difficult to explain FBI-1's growth promoting effect on HT29 cells by the mechanistic model proposed in Figure 9. During proliferation and transformation, colorectal carcinoma cells develop a variety of cellular pathways to survive. Thus, we deduced that, in HT29 cancer cells expressing mutant p53 (Arg273His), FBI-1 may act as a positive cell proliferation regulator via inhibiting tumor suppressor genes expression such as ARF or Rb.

FBI-1, encoded by Zbtb7a, is a master transcriptional repressor which belongs to the POK protein family [4], [5]. FBI-1 fulfills its transcriptional repressing roles via protein interaction mediated by its N-terminal POZ/BTB domain [3], [7], [8], [21]. Increasing evidence indicated that FBI-1 could modulates the transcription activation of several transcription factors such as p53, ER and AR [3], [7], [8], [22]. Maeda et al. 2005, [1], [6] provided evidence that FBI-1 would repress p53 activation through p19^ARF^ and MDM2 signaling pathway. FBI-1 also functions through physical interaction and co-repressors [3], [7], [8], [22]. Different from previous mechanisms data [3], [7], [8], [22], we showed that FBI-1 would also function through modulating ETS-1 activity [Fig. 9].

It is reported that ETS-1 mediates cancerous cells invasion by regulating expression of downstream genes expression, such as u-PA and MMPs. The aberrant expression of ETS-1 or MMPs is known to be poor prognostic marker [13]–[15]. Several reports also demonstrated that the u-PA and MMPs which were downstream genes of ETS-1 are involved in chemo-resistant, aggressiveness, progression and prognosis in cancer cells [23]. The ETS-1 and MMPs often aberrant express in breast cancer, lung cancer and prostate cancer [24]–[26]. ETS-1 could be activated by MAPK signaling, and it have been implicated that ETS-1 would also be a downstream effector of c-Met and HER2 signaling [27], [28]. Yu et al 2004 reported that HER2-directed therapeutic strategy, laid the foremost hope of breast cancer treatment. Studies of interaction and crosstalk of HER2 and other signaling pathways may also be useful in the anti-cancer combination therapy agent research. Our results showed that FBI-1 enhanced the transcriptional activity of ETS-1 and promoted u-PA and MMPs expression, and thereby enhancing invasion ability of Lovo cells. These suggested that FBI-1 would be a positive regulator of ETS-1 and MMPs signaling. These indicated that, FBI-1 may modulate the HER2 signaling and participate in breast cancer progress via ETS-1.

Colorectal carcinoma may lay one of the foremost threaten to human health nowadays [29]. Several signaling pathways are involved in the human colorectal carcinoma progress [29]. The p53, Ras, Myc, APC and DCC all play certain roles in colorectal carcinoma genesis [9], [10]. In these signaling pathways, Ras, Myc and APC are involved in colorectal carcinoma maintenance. Given the data that FBI-1 is a p53 negative regulator overexpressed in colon tissue, we hypothesized that FBI-1 might also be a novel molecular target of colorectal carcinoma. It is reported that p53 interacted with other transcription factor in cancerous cells. P53 and NF-kB are intertwined in cellular physiology regulation, and p53 and NF-kB repress the activities of each other [7], [9], [30]. FBI-1 down-regulates p53 expression via several mechanisms, it also enhances the transcriptional activity of NF-kB. As shown in work from Dong-Kee Lee et al., FBI-1 also functions through increasing NF-KB movement from cytoplasm into the nucleus [30]. Our results are consisted with Dong-Kee Lee et al. [30], FBI-1 promoted the cytoplasmic/nucleus translocation of ETS-1. FBI-1 enhanced the ETS-1 signaling activation, and the results would be partially reversed by p53 signaling. FBI-1 would be a novel co-regulator of ETS-1 in colorectal carcinoma cell lines, specifically through its ability to down-regulate p53 signaling.

In this work, FBI-1 promoted Lovo cells proliferation and invasion. So, FBI-1 would enhance Lovo cells proliferation, invasion and metastasis via ETS-1 signaling pathway activity. Therefore, it will be valuable to examine whether FBI-1 would be a novel regulator of ETS-1 signaling pathway. And the FBI-1 would also be the key regulator of the several signaling interaction and novel therapeutic target of human colorectal carcinoma.

## Supporting Information

File S1 **Figures S1-S4.** Figure S1. P53 knock-down or over-expression did not affect FBI-1 protein level. Figure S2. FBI-1 can enhance the recruitment of ETS-1 to the MMP9 promoter. (A) Lovo cells stably transfected with FBI-1 or empty vector were prepared and subjected to ChIP by using IgG antibody (negative control) or antibodies for ETS-1, FBI-1 and P53. The Immunoprecipitated DNA fragment was quantified by real-time PCR assay. (B) Lovo cells, which were stably transfected with FBI-1 siRNA, or control siRNA, were harvested for the ChIP assays. The ChIP assays were performed with IgG antibody (negative control) or antibodies for ETS-1, FBI-1 and P53. *P<0.05 versus the empty vector or the FBI-1 vector (A); or versus the control siRNA or the FBI-1 siRNA (B). The cloned promoter region of MMP9 is showed above the figure. Figure S3. FBI-1 can enhance the recruitment of ETS-1 to the c-Met promoter. (A) Lovo cells stably transfected with FBI-1 or empty vector were prepared and subjected to ChIP by using IgG antibody (negative control) or antibodies for ETS-1, FBI-1 and P53. The Immunoprecipitated DNA fragment was quantified by real-time PCR assay. (B) Lovo cells, which were stably transfected with FBI-1 siRNA, or control siRNA, were harvested for the ChIP assays. The ChIP assays were performed with IgG antibody (negative control) or antibodies for ETS-1, FBI-1 and P53. *P<0.05 versus the empty vector or the FBI-1 vector (A); or versus the control siRNA or the FBI-1 siRNA (B). The cloned promoter region of c-Met is showed above the figure. Figure S4. Effect of FBI-1 on ETS-1 cytoplasmic/nucleus localization. (A-D) Lovo cells were stably transfected with plasmids as indicated. (A-D) The accumulation of ETS-1 in nuclear was determined by Immunocytochemistry assays.
(DOC)
