# Correction: FBI-1 Enhances ETS-1 Signaling Activity and Promotes Proliferation of Human Colorectal Carcinoma Cells

**DOI:** 10.1371/journal.pone.0129537

**Published:** 2015-05-28

**Authors:** Min Zhu, Mingyang Li, Fan Zhang, Fan Feng, Weihao Chen, Yutao Yang, Jiajun Cui, Dong Zhang, Enqiang Linghu

The affiliation for the seventh author is incorrect. The correct affiliation is: Institute of Disease Control and Prevention, Chinese Academy of Military Medical Sciences, Beijing, P.R. China
