# Supplementary material for: FBI-1 Enhances ETS-1 Signaling Activity and Promotes Proliferation of Human Colorectal Carcinoma Cells
Source: PLoS One. 2014 May 23;9(5):e98041. doi: 10.1371/journal.pone.0098041 (PMC4032333; doi:10.1371/journal.pone.0098041)
Supplement: File S1 — Figures S1-S4. Figure S1. P53 knock-down or over-expression did not affect FBI-1 protein level. Figure S2. FBI-1 can enhance the recruitment of ETS-1 to the MMP9 promoter. (A) Lovo cells stably transfected with FBI-1 or empty vector were prepared and subjected to ChIP by using IgG antibody (negative control) or antibodies for ETS-1, FBI-1 and P53. The Immunoprecipitated DNA fragment was quantified by real-time PCR assay. (B) Lovo cells, which were stably transfected with FBI-1 siRNA, or control siRNA, were harvested for the ChIP assays. The ChIP assays were performed with IgG antibody (negative control) or antibodies for ETS-1, FBI-1 and P53. *P<0.05 versus the empty vector or the FBI-1 vector (A); or versus the control siRNA or the FBI-1 siRNA (B). The cloned promoter region of MMP9 is showed above the figure. Figure S3. FBI-1 can enhance the recruitment of ETS-1 to the c-Met promoter. (A) Lovo cells stably transfected with FBI-1 or empty vector were prepared and subjected to ChIP by using IgG antibody (negative control) or antibodies for ETS-1, FBI-1 and P53. The Immunoprecipitated DNA fragment was quantified by real-time PCR assay. (B) Lovo cells, which were stably transfected with FBI-1 siRNA, or control siRNA, were harvested for the ChIP assays. The ChIP assays were performed with IgG antibody (negative control) or antibodies for ETS-1, FBI-1 and P53. *P<0.05 versus the empty vector or the FBI-1 vector (A); or versus the control siRNA or the FBI-1 siRNA (B). The cloned promoter region of c-Met is showed above the figure. Figure S4. Effect of FBI-1 on ETS-1 cytoplasmic/nucleus localization. (A-D) Lovo cells were stably transfected with plasmids as indicated. (A-D) The accumulation of ETS-1 in nuclear was determined by Immunocytochemistry assays. (DOC) [file pone.0098041.s001.doc]

**Supplementary figure S1**

**
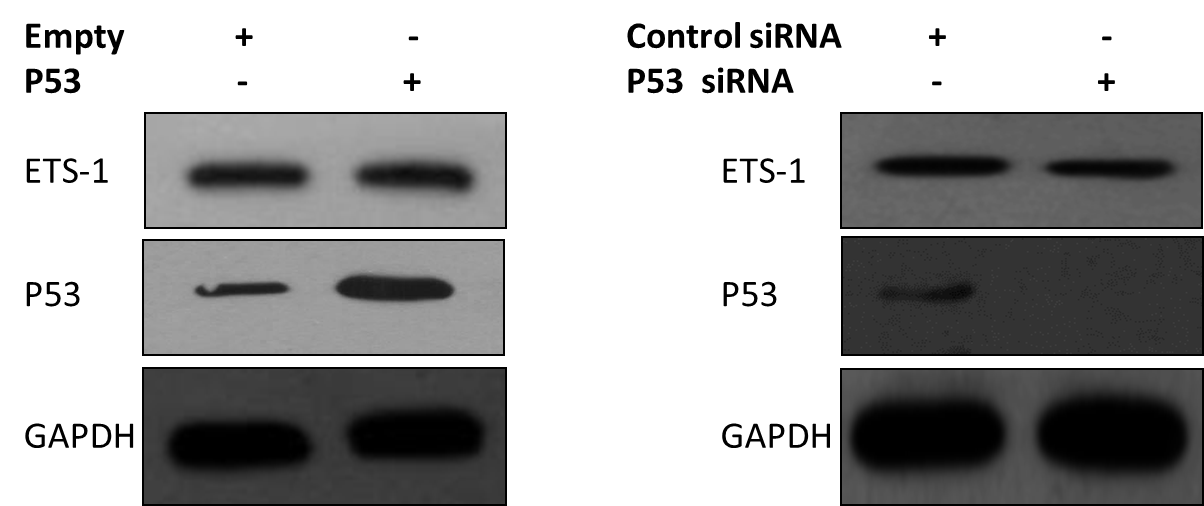
**

**Supplementary figure S2**

**
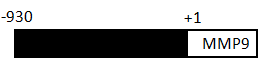
**

**
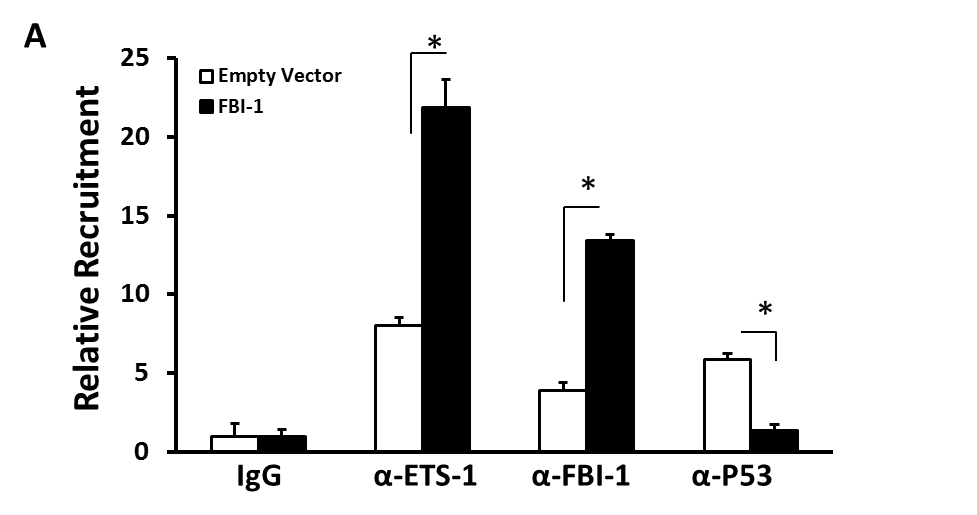
**

**
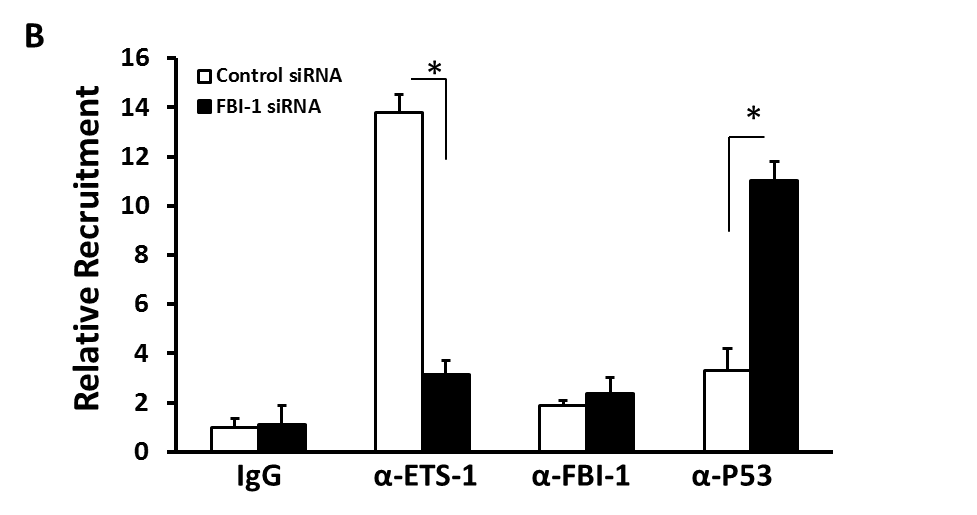
**

**Supplementary figure S3**

**
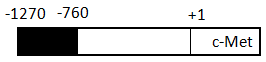
**

**
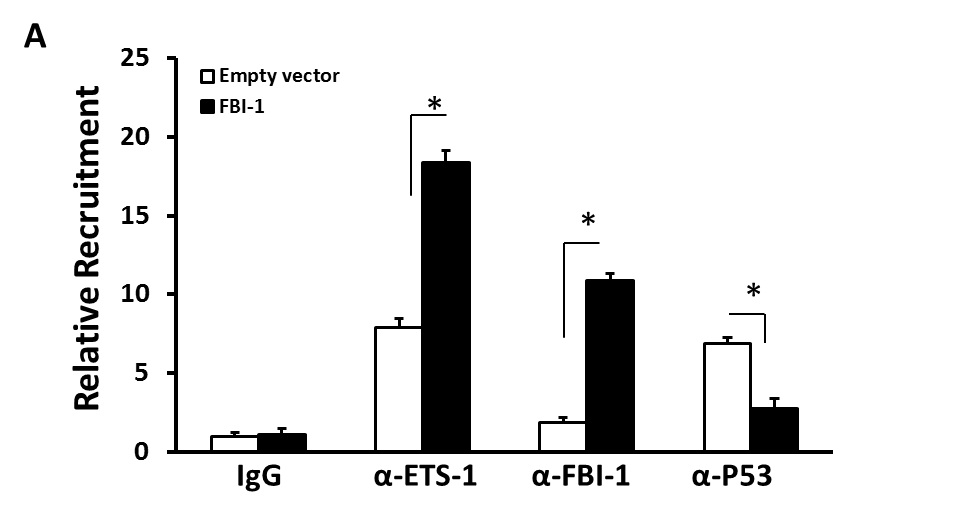
**

**
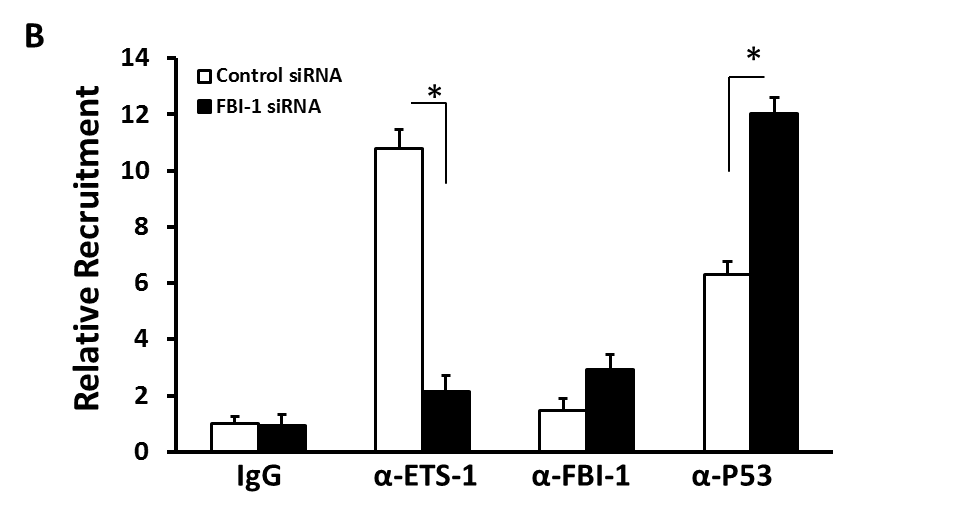
**

**Supplementary figure S4**

**
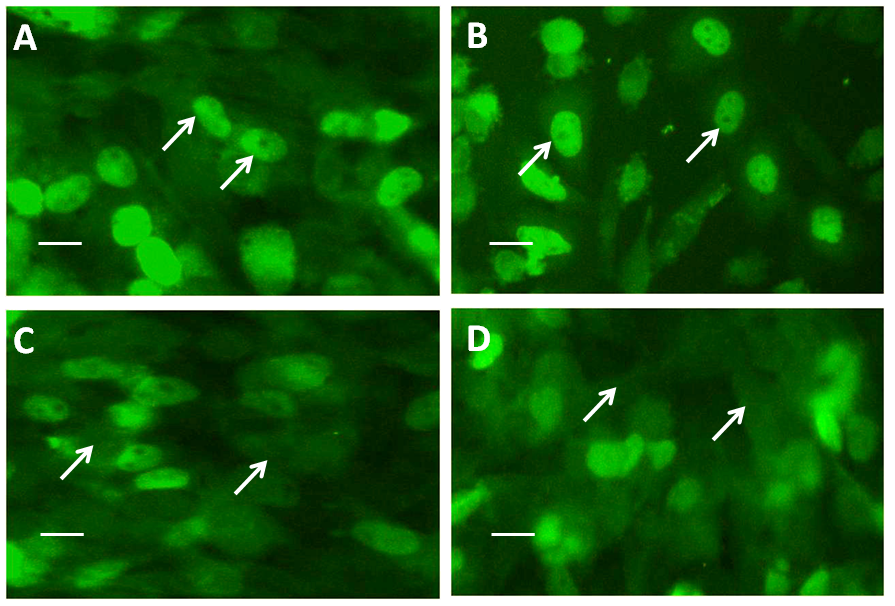
**
